# CRISPRpred: A flexible and efficient tool for sgRNAs on-target activity prediction in CRISPR/Cas9 systems

**DOI:** 10.1371/journal.pone.0181943

**Published:** 2017-08-02

**Authors:** Md. Khaledur Rahman, M. Sohel Rahman

**Affiliations:** 1 AℓEDA group, Dept. of Computer Science and Engineering, Bangladesh University of Engineering and Technology, Dhaka, Bangladesh; 2 Dept. of Computer Science and Engineering, United International University, Dhaka, Bangladesh; Indiana University, UNITED STATES

## Abstract

The CRISPR/Cas9-sgRNA system has recently become a popular tool for genome editing and a very hot topic in the field of medical research. In this system, Cas9 protein is directed to a desired location for gene engineering and cleaves target DNA sequence which is complementary to a 20-nucleotide guide sequence found within the sgRNA. A lot of experimental efforts, ranging from *in vivo* selection to *in silico* modeling, have been made for efficient designing of sgRNAs in CRISPR/Cas9 system. In this article, we present a novel tool, called CRISPRpred, for efficient *in silico* prediction of sgRNAs on-target activity which is based on the applications of Support Vector Machine (SVM) model. To conduct experiments, we have used a benchmark dataset of 17 genes and 5310 guide sequences where there are only 20% true values. CRISPRpred achieves Area Under Receiver Operating Characteristics Curve (AUROC-Curve), Area Under Precision Recall Curve (AUPR-Curve) and maximum Matthews Correlation Coefficient (MCC) as 0.85, 0.56 and 0.48, respectively. Our tool shows approximately 5% improvement in AUPR-Curve and after analyzing all evaluation metrics, we find that CRISPRpred is better than the current state-of-the-art. CRISPRpred is enough flexible to extract relevant features and use them in a learning algorithm. The source code of our entire software with relevant dataset can be found in the following link: https://github.com/khaled-buet/CRISPRpred.

## Introduction

Genome-editing technology has become very popular in recent years and it has significantly caught the sight of scientific community [1]. Rapid growth of a number of development tools makes this interesting biological phenomenon clear and helps us obtain desirable biological systems. The Clustered Regularly Inter-spaced Short Palindromic Repeats (CRISPR) and their associated endonucleas genes (Cas9) have been recently demonstrated to be a revolutionary technology for genome editing in mammalian cells [2, 3]. CRISPR/Cas9 technology functions against viral infections or other types of horizontal gene transfer by cutting down foreign DNA which attempts to spread into the host [4, 5]. This technology is easy to use which is an advantage over its genome-editing predecessors, namely, Zinc Finger Nucleases (ZFNs) and Transcription Activator Like Effector Nucleases (TALENs) [6, 7]. ZFNs and TALENs both require that scientists create custom proteins for each DNA target which requires much more effort than the painless RNA programming required for CRISPR/Cas9. Basically, Cas9 nucleases persuade double-strand breaks on targeted location of genome directed by single-guide RNAs (sgRNAs). Several studies demonstrated that the Cas9-based genome editing may introduce off-target effects which can result in a significant level of non-specific editing at other unplanned genomic loci [8, 9]. However, some recent studies have shown that the off-target effects of this technology is not as significant as previously mentioned [10, 11]. So, the efficacy and specificity of Cas9-sgRNA need to be carefully studied before applying it *in vivo*.

There are two necessary RNA components for CRISPR/Cas9 in bacteria, namely, CRISPR RNA (crRNA) and trans-activating crRNA (trRNA) which complement each other to activate and guide Cas9 nucleases [12]. A recent work suggests that a single guide RNA (sgRNA) engineering is functionally equivalent to the combining complex of both crRNA and trRNA [13]. In CRISPR/Cas9 technology, we need to design an sgRNA to have a nucleotide sequence of around 20 nucleotides which is complementary to the target region. Cas9 protein cuts off specific region of DNA (i.e., invading viral genome) guided by this rationally designed sgRNA. In a nutshell, the flexibility of RNA-guided CRISPR/Cas9 system promotes scientists to perform genome editing for virtually any locus of interest in an easy and quick way by changing the sgRNA in the expression vector. Thus efficient design of sgRNA is in great demand.

A lot of web-based tools and standalone software packages can be found in the literature for efficient designing of sgRNA in CRISPR/Cas9 technology. Some of these popular and interesting tools have been briefly reviewed below. CRISPRseek identifies gRNAs that aim at a given input sequence while minimizing off-target cleavage at other sites within any selected genome [14]. This tool can generate a cleavage score for potential off-target sequences and rank gRNAs based on the difference among predicted cleavage scores in given input sequence. CHOPCHOP also uses scoring functions, efficient sequence alignment algorithms to minimize search times, and rigorously predicts off-target binding of sgRNAs [15]. CasOT and Cas-OFFinder search potential off-target sites for any given input genome sequence with given types of Protospacer Adjacent Motif (PAM), and the number of mismatches allowed in the seed and non-seed regions [16, 17]. CCTop is a web-based tool that reports and orders all possible sgRNAs based on off-target quality and shows full documentation [18]. The CRISPRscan algorithm designs highly efficient sgRNAs for genome editing analyzing different types of molecular features such as guanine enrichment, adenine depletion, nucleosome positioning, etc.; it also analyzes secondary structures of sgRNAs to extract some thermodynamic features [19]. The sgRNAcas9 software package searches for CRISPR target sites based on given parameters and minimizes off-target effects [20]. WU-CRISPR analyzes RNA-seq data and identify some novel features that contribute to designing highly efficient sgRNAs [21]. CRISPR-ERA tool designs efficient sgRNAs specifically for gene editing, repression and activation [22]. The CRISPR Design tool reports that high concentrations of the enzyme increase off-site target effects, whereas lower concentration of Cas9 increases specificity while reducing on-target cleavage activity [8]. Chari et al. have developed sgRNA Scorer tool to assess sgRNA activity across 1,400 genomic loci [23]. Their unraveled underlying nucleotide sequence and epigenetic parameters have contributed to designing an improved mechanism of genome targeting. SSC, RDHA sgRNA and CRISPy-web are some online tools for the design of highly active sgRNAs for any gene of interest [10, 24, 25]. Azimuth is another important tool for *in silico* modeling of sgRNAs activity which is currently the state-of-the-art for on-target prediction [26]. Most of these tools analyze the composition of nucleotides of sgRNAs in animals and report regarding nucleotide preferences. However, a recent study of plant suggests that there is no such nucleotide preferences in plant sgRNAs [27].

**Our Contributions:** On-target activity prediction tools are very important for genome editing. High predictive performance in on-target activity of sgRNAs is highly demanding for CRISPR/Cas9 systems. In this article, we present a new tool, namely, CRISPRpred that predicts on-target activity of *in-silico* sgRNAs efficiently. Our overall contributions are as follows:

- We present a generalized method that produces all possible position specific features and sequence-based features. In addition, our method also produces secondary structure related features.
- Our method extracts relevant features based on the importance scores of random forest. After that a single pass *anova* test keeps relevant features based on *p*-value.
- We train a Support Vector Machine (SVM) to build a learning model and conduct experiments on publicly available dataset using relevant features that have importance scores above a threshold.
- Finally, we compare experimental results of CRISPRpred with Azimuth which is currently the state-of-the-art and show the effectiveness and efficacy of the newly introduced tool.

## Results

It has been observed in the literature that the mutagenic performance of CRISPR/Cas9 system differs significantly due to a small change in sgRNAs [8]. Mandal et al. has suggested that the on-target performance of site-directed mutation is greatly dependent on the sgRNA provided that sgRNAs targeting the same genomic locus show various activities [28]. As discussed earlier, a handful of studies have focused on sequence determinants of sgRNAs to predict cleavage activity [21, 24, 26]. Ma et al. have suggested that the secondary structure of sgRNAs may contribute to editing efficiency [29]. In our work, we have focused on both position-specific sequences of adjacent nucleotides and secondary structures to construct features from sgRNAs. We have re-analyzed a public dataset of 5310 guide sgRNAs (see S1 File) in total for feature extraction and selection that are highly associated with CRISPR activities.

We have conducted experiments and found that CRISPRpred outperforms Azimuth. It is also important to note that choosing a suitable evaluation metric is equally important like using a suitable method. We have analyzed Receiver Operating Characteristic Curve (ROC-Curve), Area Under Precision-Recall Curve (AUPR-Curve), Sensitivity-Specificity Curve (SS-Curve), Matthews Correlation Coefficient Curve (MCC-Curve) and Root Mean Square Error (RMSE) to compare the results [30, 31].

### Experimental setup

**Fig 1:**
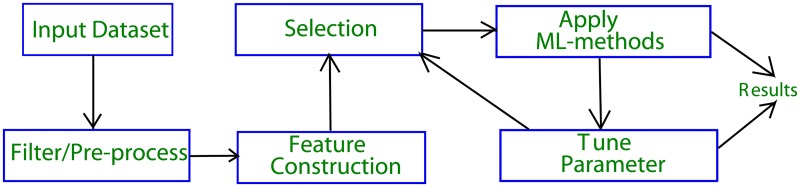
Workflow of CRISPRpred. In the first step, input dataset is preprocessed to discard any kind of irrelevant information. In the next step, it constructs features based on position-specific sequence, position-independent sequence and secondary structures. In extraction and selection step, a feature selection algorithm is run to select top relevant features. In the next step, SVM is applied in ‘Apply ML-methods’ stage to check the performance. We can also tune different parameters of feature selection algorithms and apply SVM again. In each case, we note important results.

We have used three different machines to conduct the experiments, namely, a Desktop computer with Intel Core i3 CPU @ 1.90GHz x 4, Ubuntu 15.10 64-bit OS and 4 GB RAM, a Desktop computer with Intel Core i7 CPU @ 3.30GHz x 4, Windows 7, 64-bit OS and 8 GB RAM, and a Dell PowerEdge R430 Rack Server with Ubuntu 13.04 OS, Intel^®^ Xeon^®^ E5-2620 v4 2.1GHz, 20M Cache and 8GB RDIMM. We have used R language version 3.2.1. In ‘Methods’ section, we have reported all other packages used in our experiments. A procedural flow of our experiments has been shown in Fig 1. In the dataset, there can be some irrelevant information like name of target gene, name of drug and others that are not useful for making prediction. We have discarded those irrelevant information in the preprocessing step. Generally, a ‘score’ is calculated based on how successful a guide sequence is at knocking out its target gene and we do not need to process target gene name after calculating the score. We also filter and preprocess such information from the input dataset. Then, we construct features using our tool as described in the ‘Methods’ sections. After feature construction, we keep all those information in the dataset that are relevant and supposed to be important for the SVM. In the next step, we run random forest algorithm to select the most relevant features. Some features may not contain any information for prediction. For example, we constructed a binary feature that indicates the position of ‘T’ in 26*^th^* place in a 30-mer sgRNA; but it does not carry any significant information to predict the activity well. Such features have also been discarded after running the selection process and only relevant features are retained for the next step. We apply SVM on the dataset for different combinations of finally selected features. Different parameters can be tuned for random forest and SVM, e.g., learning rate in SVM, number of trees in random forest, etc. and we also record the results. For each setup of parameters, we run each algorithm 30 times and report the average results. We compute the evaluation metrics using leave-one-gene-out cross-validation.

### Feature construction, importance and selection

A plethora of previous studies has shown that both sequence characteristics and structural characteristics of sgRNAs play important roles in target cleavage. So, in this research, we have explored positions of nucleotides as well as secondary structures of sgRNAs to construct features (see ‘Methods’ section). In addition, some factors can trigger a particular chain of events that we need to study as well [32]. Position specific features are related to positions of nucleotides in sgRNAs and also affect the mutagenic activity. These may include specific position of a single nucleotide in an sgRNA, specific position of a pair of nucleotides, specific position of three consecutive nucleotides, etc. Structural features, also known as thermodynamic properties, include Minimum Free Energy (MFE), most favorable thermodynamic RNA-RNA interaction energy, local pair probabilities and specific heat of the corresponding 30-mer of sgRNAs. There are also some features that are treated as position independent features and are not related to previous types such as GC count, i.e., how many times G and C appear in a 30-mer guide; AT count, i.e., how many times AT pair appears in a 30-mer guide and A count, i.e., how many times A appears in a 30-mer guide. Besides these, we have used two features found in the dataset that also improve the predictive performance. The name of those two features are ‘amino acid cut position’ and ‘percent peptide’. Detail information about the dataset can be found in [26]; however, we have collected the dataset from Azimuth website [33].

**Fig 2:**
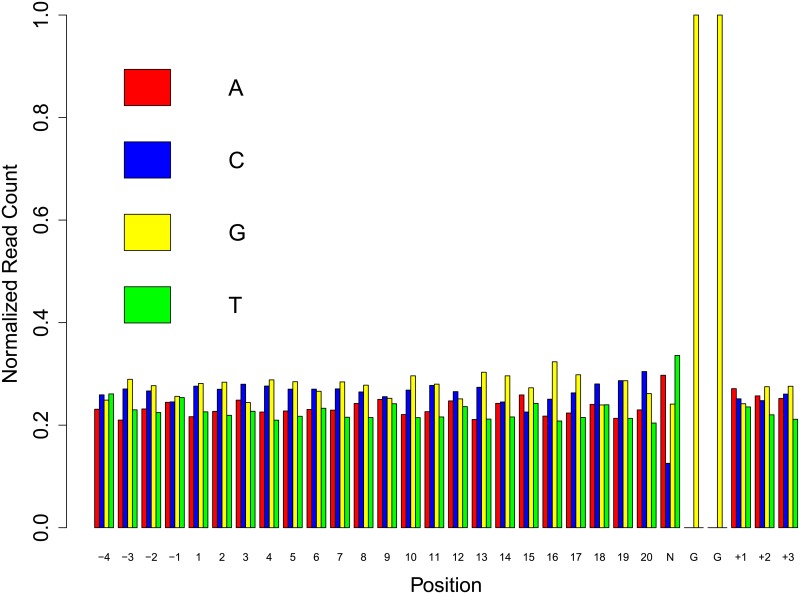
Normalized read count of ‘A’, ‘C’, ‘G’ and ‘T’ in 30-mer sgRNAs. ‘N’ represents any nucleotide.

**Fig 3:**
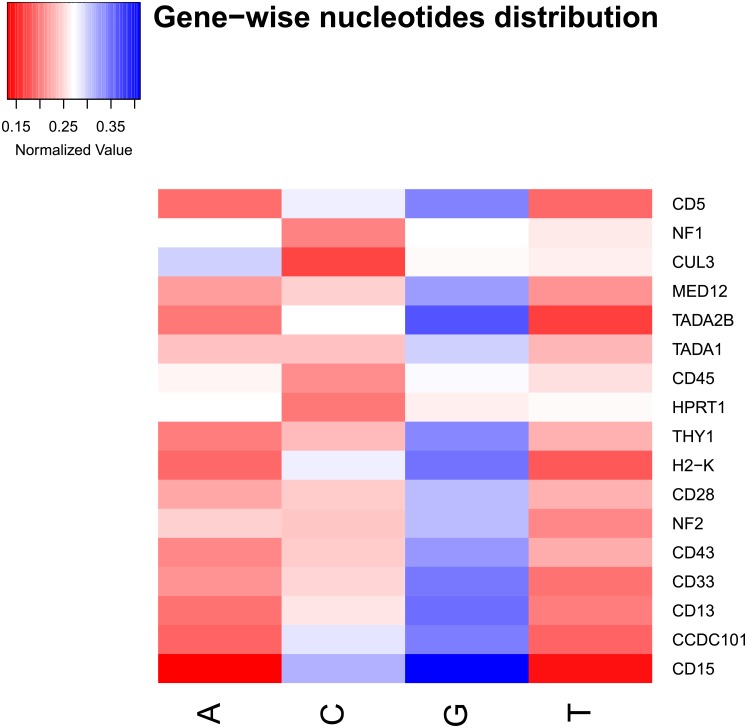
Gene-wise frequency distribution of nucleotides for all sgRNAs. All genes are enriched with ‘G’ except ‘CUL3’.

We have reported the normalized read count of single nucleotide position specific features from 30-mer sgRNAs in Fig 2. We see that the 25*^th^* position has any nucleotide followed by two G’s in the 26*^th^* and 27*^th^* positions. Here, the motif “NGG” is the Protospacer Adjacent Motif (PAM), where ‘N’ is any nucleotide of ‘A’, ‘C’, ‘G’ or ‘T’ [8]. From gene specific frequency distribution of all 30-mer sgRNAs, we can deduce that most of the genes are enriched with ‘G’ in 30-mer sgRNAs (see Fig 3). So ‘G’ enrichment is likely to be an important feature. Thus, normalized read count and gene specific nucleotides distribution give us the idea of position specific features and position independent features, respectively.

**Fig 4:**
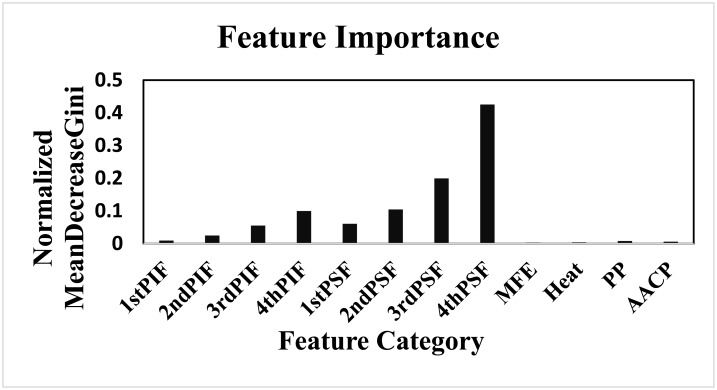
Normalized importance values (Mean Decrease Gini of Random Forest) of different category-wise features. ‘1stPIF’, ‘2ndPIF’, ‘3rdPIF’ and ‘4thPIF’ represent category-wise summation of importance values of position independent features where first three character of each category-name denotes the number of nucleotides appear together in 30-mer sgRNAs; e.g., ‘3rdPIF’ represents summation of importance value for all position independent features of three consecutive nucleotides (tri-nucleotides). Similarly, ‘1stPSF’, ‘2ndPSF’, ‘3rdPSF’ and ‘4thPSF’ represent category-wise summation of importance values of position specific features where first three character of each category-name denotes the number of nucleotides appear together in 30-mer sgRNAs; e.g., ‘4rdPSF’ represents summation of importance value for all position specific features where four consecutive nucleotides (tetra-nucleotides) are considered. Thermodynamic features ‘MFE’ and ‘Heat’ mean minimum free energy and heat of 30-mer sgRNAs, respectively. ‘PP’ represents percentage of peptide and ‘AACP’ represents amino acid cut position.

All features do not carry the same information to build an efficient prediction model. Thus we need to select important features and discard all irrelevant features. There are various existing machine learning methods such as wrapper or filter method that we can apply to do this job. In this work, we have used random forest algorithm for feature selection based on importance scores (Mean Decrease Gini) where we set a threshold to select all relevant features. We have shown category-wise feature importance based on normalized Mean Decrease Gini of Random Forest in Fig 4. Here, single-nucleotide, di-nucleotides, tri-nucleotides and tetra-nucleotides position specific features are referred to as follows: first three character of each category-name denotes the number of nucleotides appear together in 30-mer sgRNAs; e.g., ‘3rdPIF’ represents summation of importance scores for all position independent features of three consecutive nucleotides (tri-nucleotides).

There are some features like ‘G_24’ and ‘GAGG_24’ with ‘G’ in the 24*^th^* position in both cases. Intuitively, one may think that these two features may have no combined effect, i.e., if we remove any one of these features from the feature list then performance metrics will not change. To investigate this issue, we have conducted experiments using linear regression model with 10-fold cross-validation keeping both of these features along with all other features and recorded Root Mean Square Error (RMSE). Then, we have excluded ‘GAGG_24’ from the feature list and observed that RMSE decreases. This experiment validates that ‘G_24’ and ‘GAGG_24’ position specific features have some combined effect along with all other selected features.

**Fig 5:**
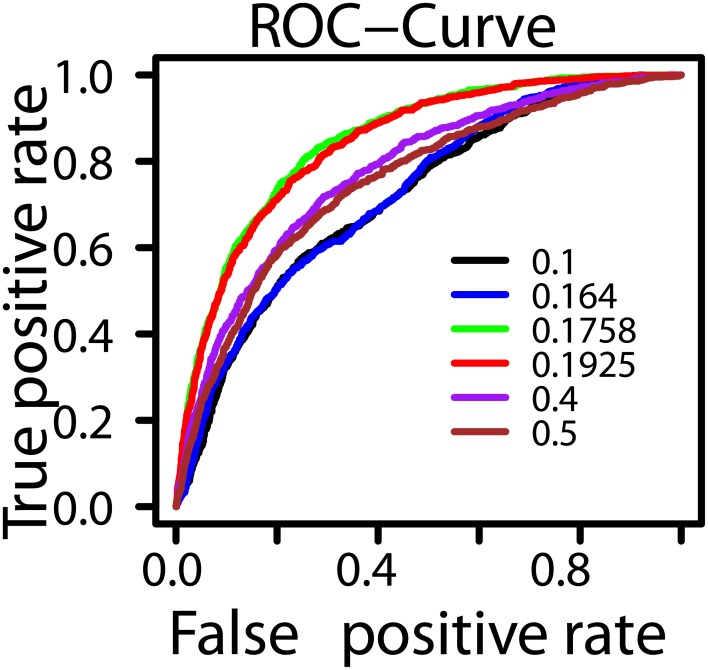
Comparison among ROC-Curves for different thresholds values of importance scores. For each threshold, we build a linear formula taking corresponding features and train dataset. Then, we perform SVM method based on the formula and check prediction values to draw ROC-Curves. In all experiments, we keep the same parametric values and perform leave-one-gene-out cross-validation.

Another interesting finding is that though we extract 9632 features (see S2 File) in total, we can not use some features for training that have importance values greater than zero. This is because we have only 5310 guide sequences to train and test. If the size of features is greater than the size of dataset, then SVM does not work well. Therefore, we do these experiments setting some thresholds for importance values. We have shown different threshold values and corresponding ROC-Curves in Fig 5. When an ROC-curve gets the left top corner of the graph then it indicates better performance [30]. To conduct experiments, we first set the threshold value to 0.5 and draw the ROC-Curve. Then, we decrease threshold value to 0.4 and draw the corresponding ROC-Curve. In this case, we see that the number of selected features is more than the previous case and performance is also better. We get similar results when we set the threshold to 0.19 and 0.18. But, when we decrease more (i.e., reach 0.17 and 0.1), the performance degrades. This happens presumably because, in this case, the total number of features actually crosses a limit which in some sense is incompatible/unreasoanble with respect to the size of the datasets (i.e., 5310). So, if we get a dataset where 9632 features are reasonable, then we can expect to get even better predictive performance. We have summarized the results in Table 1 (highest values are shown in bold face). We see that CRISPRpred performs better when we set threshold to 0.18.

**Table 1:** Threshold of importance score, corresponding total number of features, AUROC-Curve and AUPR-Curve.

Threshold of Importance Score	Number of Features	AUROC	AUPR
0.1	4790	0.72	0.36
0.17	3120	0.73	0.37
0.18	2899	**0.85**	**0.56**
0.19	2609	0.84	0.56
0.4	890	0.78	0.46
0.5	606	0.75	0.43

### Evaluation and comparison

**Fig 6:**
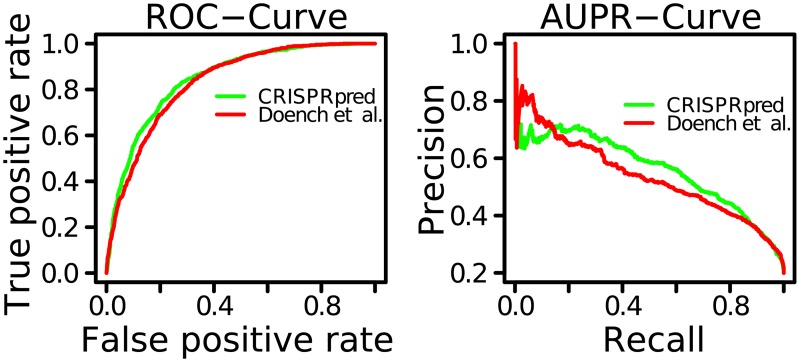
Comparison between CRISPRpred and Azimuth (labeled as Doench et al.). Left figure shows comparison of ROC-Curve and right figure shows comparison of Area Under PR-Curve (AUPR-Curve).

**Fig 7:**
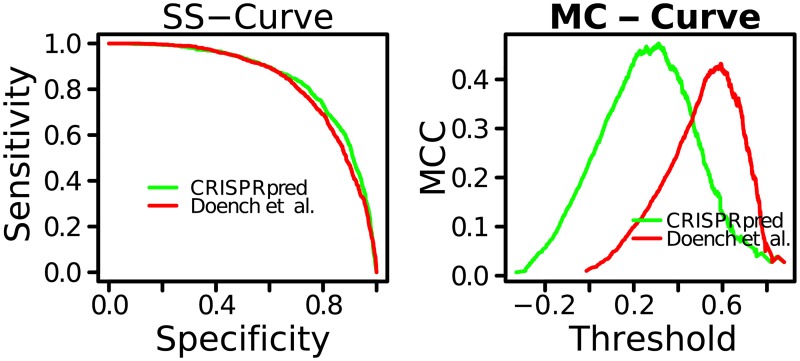
Comparison between CRISPRpred and Azimuth (labeled as Doench et al.). Left figure shows comparison of Sensitivity Specificity Curve (SS-Curve) and right figure shows comparison of Matthews Correlation Coefficient Curve (MCC-Curve).

Once we have selected relevant position independent features and position-specific features, we are ready to evaluate the predictive performance of CRISPRpred. We also compare the results with current state-of-the-art method (Azimuth). We have used linear combination of all features for CRISPRpred. We have set default parameters for SVM and we choose threshold value for importance score as 0.18 (Please see Table 1). Unless otherwise noted, we have performed leave-one-gene-out cross-validation over the dataset. Note that, we have restricted the parametric values of all methods to avoid over-fitting, i.e., same parametric values have been used for both training and testing cases. We have reported the results in Figs 6 and 7. We have conducted the *anova* test on linear regression model and found that some features like ‘AC_25’ and ‘T_26’ are redundant. So, we have excluded those features from the feature list and conducted the experiment again to record RMSE. Interestingly, we have found that there is no change in the RMSE and other performance metrics, i.e., those features are indeed redundant. In this way, we deduce that active sgRNA is ‘G’ rich which is supported by CRISPRscan [34]. Our experimental results show that stable sgRNA is ‘A’ rich (*p*-value = 1.036413e-78) which is also supported by Azimuth [33].

We have reported the results of Receiver Operating Characteristics (ROC) curves and Precision-Recall (PR) curves in Fig 6. When ROC-curve gets the left upper corner, then it indicates better performance and when the PR-curve gets the right upper corner of the graph then it shows better performance [30, 31]. Actually, in those cases, we get higher value for area under ROC-curve or area under PR-curve. In Fig 6, we see that CRISPRpred beats Azimuth (labeled as Doench et al. [26]) in both ROC-Curve analysis and AUPR-Curve analysis. The values of area under ROC-Curve (AUROC-Curve) for CRISPRpred and Azimuth are 0.85 and 0.83, respectively. In the dataset (*FC*_*plus*_*RES*.*csv*) provided in Azimuth website [33], there are only 20% true values. In such case, AUPR-Curve analysis explains better and we perform this experiment also. We find the values of area under PR-Curve (AUPR-Curve) for CRISPRpred and Azimuth are 0.56 and 0.53, respectively. Clearly, we get approximately 2% improvement in AUROC-Curve and 5% improvement in AUPR-Curve that are very important for CRISPR/Cas9 systems where higher accuracy is highly demanding.

We compare some other metrics in Fig 7. In Sensitivity Specificity Curve (SS-Curve) and Matthews Correlation Coefficient Curve (MCC-Curve), CRISPRpred also performs better than Azimuth. Maximum MCC values for CRISPRpred and Azimuth are 0.48 and 0.43, respectively. We also find CRISPRpred performing better here. We have labeled predictions of CRISPRpred that can be found with the software (see S3 File).

### Limitations

We have examined significantly on *in silico* predictions, i.e., only on-target prediction has been considered but off-target effects are also worth mentioning for erroneous sgRNAs. To this end, we have conducted a narrow set of experiments where we have used CCtop site [35] to check off-target effects. The results are not mentionable now; however, we plan to work on it with broader datasets and integrate with CRISPRpred in future to get the best performance for both on-target and off-target predictions.

### Conclusion

In this article, our investigation provides two key factors that improve the in silico prediction of sgRNA activity in CRISPR/Cas9 system. First of all, we have incorporated all possible single, di-nucleotides, tri-nucletides and tetra-nucleotides position specific features and position independent features. Our newly introduced tool has definitely increased predictive performance. Secondly, we have examined that in addition to ‘G’-rich property, active sgRNA is enriched with ‘A’ (*p*-value = 1.036413e-78) but is ‘T’ depleted. We have selected relevant features to build CRISPRpred that consists of SVM algorithm along with some other functionalities. In our experiments, for CRISPRpred, we have achieved excellent values of 0.85, 0.56 and 0.48 for AUROC-Curve, AUPR-curve and maximum MCC, respectively. In all aspects of on-target prediction, CRISPRpred performs better than the current state-of-the-art.

## Methods

### Dataset

We have used a combination of two primary datasets available in [10], which we refer to as “FC-RES” because the methods used to detect successful knockdowns were Flow Cytometry (FC) and RESistance assays. Notably, both FC and RES datasets are also separately available. The FC-RES dataset consists of 17 genes and 4380 unique guides targeting 11 human genes (CD13, CD15, CD33, CCDC101, MED12, TADA2B, TADA1, HPRT, CUL3, NF1, NF2) and 6 mouse genes (Cd5, Cd28, H2-K, Cd45, Thy1, Cd43) (see S1 File). Unless otherwise noted, while conducting experiments, we have always used one-gene-out cross-validation to see the effectiveness of our tool. More details about the dataset can be found in [26] and [10]. A summary of the dataset has been discussed at the beginning of results section. We have re-analyzed the dataset using our data manipulation code and Vienna package [36] to identify novel features that are correlated to sgRNA efficacy.

### Machine learning algorithms

We have incorporated Linear Regression (LR), Random Forest (RF) and SVM machine learning algorithms to conduct experiments. We have used linear kernel function with SVM. We have also used evaluation metrics for binary classifier. We have written code in R language for each of these models. For all these algorithms, we have used **e1071**, **randomForest**, **ROCR**, **caTools** R packages. While conducting experiments, we have performed leave-one-gene-out cross-validation. We allow a maximum number of trees to be 500 while conducting experiments for the RF model. We have observed that predictive ability of the SVM model increases with the increase in the number of relevant features.

### Features

We have constructed features from sgRNA guides using a python program which is available in the software package. We have treated each position of a nucleotide as a binary value and deduced 120 features. For example, we get 4 features for the first position of a sgRNA based on the presence of any of four nucleotides (i.e., any of A, C, G, T) and there are 30 positions in 30-mer sgRNAs. Then we have created another 464 binary features by checking whether there are two same consecutive nucleotides in the first and second positions of a sgRNA, respectively. For example, if first nucleotide is ‘A’ and second nucleotide is ‘A’ in a sgRNA then we treat this as 1, otherwise we simply put a 0 which means the absence of ‘AA’ in the first and second positions in sgRNA. We have also mixed two types of nucleotides that are adjacent and repeated the previous feature construction process to get more binary features. For example, we get a feature considering that the first nucleotide is ‘A’ and the second nucleotide is ‘C’ in 30-mer sgRNAs. Similarly, we get another feature if first nucleotide is ‘C’ and second nucleotide is ‘A’. In this way, we get 29 features for one type of nucleotide and a total of 29 × (4)^2^ = 464 features. Then we have mixed three types of nucleotides that are adjacent and repeated the previous feature construction process again to consider 28 × (4)^3^ = 1792 more features. We have also mixed four types of nucleotides that are adjacent and repeated the previous feature construction process again to consider 27 × (4)^4^ = 6912 more features. In addition to these, we have deduced position independent features like GC content, AT content, A content, C content, G content, T content, etc. We get four position independent features considering single nucleotide, namely, A, C, G and T. After that we get 16 position independent features considering different combinations of two nucleotides (e.g., AA, AC, AG, etc.). Similarly, we also consider different combinations of three nucleotides and four nucleotides. Finally, we get a total 4^1^ + 4^2^ + 4^3^ + 4^4^ = 340 position independent features. We get two important features, namely, amino acid cut position, and percent peptide from the dataset provided in the Azimuth website.

We have constructed thermodynamic features using ViennaRNA Package version 2.0 [37]. We have calculated Minimum Free Energy (MFE) for each 30-mer of sgRNAs using RNAfold with default parameters and interpreted this as a feature. We have also calculated specific heat of corresponding 30-mer of sgRNAs using RNAheat.

Thus, we have extracted a total of 120 + 464 + 1792 + 6912 + 340 + 2 + 2 = 9632 features. After considering all these features, we have performed a wrapper algorithm built around random forest [38] to select relevant features. We have used **Boruta** package of R language for this purpose. **Boruta** function reports any of three states (‘Confirmed’, ‘Tentative’ and ‘Rejected’) for all features. All confirmed features are relevant and we have ranked them based on importance. We have also used **randomForest** package for the same purpose and found that this method is more suitable than **Boruta**. Finally, we have used relevant features to train the machine learning algorithms.

### Statistical significance

We have evaluated statistical significance by performing an *anova* test. We have also analyzed Root Mean Square Error (RMSE) after appending each statistically significant feature to the feature list. We have used our customized R programs for various estimations that are available online.

### Software

All of our source code, experimental results, data manipulation code, dataset and figure generation code are available at the following link: https://github.com/khaled-buet/CRISPRpred

## Supporting information

S1 File Dataset. A public dataset that contains 5310 guide sgRNAs.
(CSV)

S2 File Extracted features. This file contains 9632 extracted features.
(CSV)

S3 File Prediction score. This file contains scores predicted by CRISPRpred.
(CSV)

## References

[1] Doudna JA, Charpentier E. The new frontier of genome engineering with CRISPR-Cas9. Science. 2014;346(6213):1258096 10.1126/science.1258096 25430774

[2] Cong L, Ran FA, Cox D, Lin S, Barretto R, Habib N, et al Multiplex genome engineering using CRISPR/Cas systems. Science. 2013;339(6121):819–823. 10.1126/science.1231143 23287718 PMC3795411

[3] Mali P, Yang L, Esvelt KM, Aach J, Guell M, DiCarlo JE, et al RNA-guided human genome engineering via Cas9. Science. 2013;339(6121):823–826. 10.1126/science.1232033 23287722 PMC3712628

[4] Brouns SJ, Jore MM, Lundgren M, Westra ER, Slijkhuis RJ, Snijders AP, et al Small CRISPR RNAs guide antiviral defense in prokaryotes. Science. 2008;321(5891):960–964. 10.1126/science.1159689 18703739 PMC5898235

[5] Marraffini LA, Sontheimer EJ. CRISPR interference limits horizontal gene transfer in staphylococci by targeting DNA. science. 2008;322(5909):1843–1845. 10.1126/science.1165771 19095942 PMC2695655

[6] Urnov FD, Miller JC, Lee YL, Beausejour CM, Rock JM, Augustus S, et al Highly efficient endogenous human gene correction using designed zinc-finger nucleases. Nature. 2005;435(7042):646–651. 10.1038/nature03556 15806097

[7] Mussolino C, Morbitzer R, Lütge F, Dannemann N, Lahaye T, Cathomen T. A novel TALE nuclease scaffold enables high genome editing activity in combination with low toxicity. Nucleic acids research. 2011;39(21):9283–9293. 10.1093/nar/gkr597 21813459 PMC3241638

[8] Hsu PD, Scott DA, Weinstein JA, Ran FA, Konermann S, Agarwala V, et al DNA targeting specificity of RNA-guided Cas9 nucleases. Nature biotechnology. 2013;31(9):827–832. 10.1038/nbt.2647 23873081 PMC3969858

[9] Pattanayak V, Lin S, Guilinger JP, Ma E, Doudna JA, Liu DR. High-throughput profiling of off-target DNA cleavage reveals RNA-programmed Cas9 nuclease specificity. Nature biotechnology. 2013;31(9):839–843. 10.1038/nbt.2673 23934178 PMC3782611

[10] Doench JG, Hartenian E, Graham DB, Tothova Z, Hegde M, Smith I, et al Rational design of highly active sgRNAs for CRISPR-Cas9-mediated gene inactivation. Nature biotechnology. 2014;32(12):1262–1267. 10.1038/nbt.3026 25184501 PMC4262738

[11] Ho TT, Zhou N, Huang J, Koirala P, Xu M, Fung R, et al Targeting non-coding RNAs with the CRISPR/Cas9 system in human cell lines. Nucleic acids research. 2014;43(3):e17–e17. 10.1093/nar/gku1198 25414344 PMC4330338

[12] Barrangou R, Fremaux C, Deveau H, Richards M, Boyaval P, Moineau S, et al CRISPR provides acquired resistance against viruses in prokaryotes. Science. 2007;315(5819):1709–1712. 10.1126/science.1138140 17379808

[13] Jinek M, Chylinski K, Fonfara I, Hauer M, Doudna JA, Charpentier E. A programmable dual-RNA–guided DNA endonuclease in adaptive bacterial immunity. Science. 2012;337(6096):816–821. 10.1126/science.1225829 22745249 PMC6286148

[14] Zhu LJ, Holmes BR, Aronin N, Brodsky MH. CRISPRseek: a bioconductor package to identify target-specific guide RNAs for CRISPR-Cas9 genome-editing systems. PLoS One. 2014;9(9):e108424 10.1371/journal.pone.0108424 25247697 PMC4172692

[15] Montague TG, Cruz JM, Gagnon JA, Church GM, Valen E. CHOPCHOP: a CRISPR/Cas9 and TALEN web tool for genome editing. Nucleic acids research. 2014; p. gku410. 10.1093/nar/gku410 PMC4086086 24861617

[16] Xiao A, Cheng Z, Kong L, Zhu Z, Lin S, Gao G, et al CasOT: a genome-wide Cas9/gRNA off-target searching tool. Bioinformatics. 2014;30(8):1180–1182. 10.1093/bioinformatics/btt764 24389662

[17] Bae S, Park J, Kim JS. Cas-OFFinder: a fast and versatile algorithm that searches for potential off-target sites of Cas9 RNA-guided endonucleases. Bioinformatics. 2014;30(10):1473–1475. 10.1093/bioinformatics/btu048 24463181 PMC4016707

[18] Stemmer M, Thumberger T, del Sol Keyer M, Wittbrodt J, Mateo JL. CCTop: an intuitive, flexible and reliable CRISPR/Cas9 target prediction tool. PloS one. 2015;10(4):e0124633 10.1371/journal.pone.0124633 25909470 PMC4409221

[19] Moreno-Mateos MA, Vejnar CE, Beaudoin JD, Fernandez JP, Mis EK, Khokha MK, et al CRISPRscan: designing highly efficient sgRNAs for CRISPR-Cas9 targeting in vivo. Nature methods. 2015;12(10):982–988. 10.1038/nmeth.3543 26322839 PMC4589495

[20] Xie S, Shen B, Zhang C, Huang X, Zhang Y. sgRNAcas9: a software package for designing CRISPR sgRNA and evaluating potential off-target cleavage sites. PloS one. 2014;9(6):e100448 10.1371/journal.pone.0100448 24956386 PMC4067335

[21] Wong N, Liu W, Wang X. WU-CRISPR: characteristics of functional guide RNAs for the CRISPR/Cas9 system. Genome biology. 2015;16(1):1–8. 10.1186/s13059-015-0784-0 26521937 PMC4629399

[22] Liu H, Wei Z, Dominguez A, Li Y, Wang X, Qi LS. CRISPR-ERA: a comprehensive design tool for CRISPR-mediated gene editing, repression and activation. Bioinformatics. 2015; p. btv423. 10.1093/bioinformatics/btv423 PMC4757951 26209430

[23] Chari R, Mali P, Moosburner M, Church GM. Unraveling CRISPR-Cas9 genome engineering parameters via a library-on-library approach. Nature methods. 2015;12(9):823–826. 10.1038/nmeth.3473 26167643 PMC5292764

[24] Xu H, Xiao T, Chen CH, Li W, Meyer C, Wu Q, et al Sequence determinants of improved CRISPR sgRNA design. Genome research. 2015; p. gr–191452. 10.1101/gr.191452.115 PMC4509999 26063738

[25] Blin K, Pedersen LE, Weber T, Lee SY. CRISPy-web: An online resource to design sgRNAs for CRISPR applications. Synthetic and Systems Biotechnology. 2016;. 10.1016/j.synbio.2016.01.003 PMC5640694 29062934

[26] Doench JG, Fusi N, Sullender M, Hegde M, Vaimberg EW, Donovan KF, et al Optimized sgRNA design to maximize activity and minimize off-target effects of CRISPR-Cas9. Nature biotechnology. 2016;. 10.1038/nbt.3437 26780180 PMC4744125

[27] Liang G, Zhang H, Lou D, Yu D. Selection of highly efficient sgRNAs for CRISPR/Cas9-based plant genome editing. Scientific reports. 2016;6. 10.1038/srep21451 PMC4759811 26891616

[28] Mandal PK, Ferreira LM, Collins R, Meissner TB, Boutwell CL, Friesen M, et al Efficient ablation of genes in human hematopoietic stem and effector cells using CRISPR/Cas9. Cell stem cell. 2014;15(5):643–652. 10.1016/j.stem.2014.10.004 25517468 PMC4269831

[29] Ma X, Zhang Q, Zhu Q, Liu W, Chen Y, Qiu R, et al A robust CRISPR/Cas9 system for convenient, high-efficiency multiplex genome editing in monocot and dicot plants. Molecular plant. 2015;8(8):1274–1284. 10.1016/j.molp.2015.04.007 25917172

[30] Fawcett T. An introduction to ROC analysis. Pattern recognition letters. 2006;27(8):861–874. 10.1016/j.patrec.2005.10.010

[31] Davis J, Goadrich M. The relationship between Precision-Recall and ROC curves. In: Proceedings of the 23rd international conference on Machine learning. ACM; 2006. p. 233–240.

[32] Guyon I, Elisseeff A. An introduction to variable and feature selection. The Journal of Machine Learning Research. 2003;3:1157–1182.

[33] Azimuth Website; Last accessed on 2nd July, 2017, 10:00 AM. Available from: http://research.microsoft.com/en-us/projects/azimuth/.

[34] CRISPRscan Website; Last accessed on 2nd July, 2017, 10:00 AM. Available from: http://www.crisprscan.org/.

[35] CCtop Website; Last accessed on 2nd July, 2017, 10:00 AM. Available from: http://crispr.cos.uni-heidelberg.de/.

[36] Lorenz R, Bernhart SH, Zu Siederdissen CH, Tafer H, Flamm C, Stadler PF, et al ViennaRNA Package 2.0. Algorithms for Molecular Biology. 2011;6(1):1 10.1186/1748-7188-6-26 22115189 PMC3319429

[37] ViennaRNA Package 2.0; Last accessed on 2nd July, 2017, 10:00 AM. Available from: http://www.tbi.univie.ac.at/RNA/.

[38] Kursa MB, Rudnicki WR, et al Feature Selection with the Boruta Package. Journal of Statistical Software;36(i11).

